# A programme evaluation of ‘First Steps’: A peer-conceived, developed and led self-management intervention for people after a Parkinson's diagnosis

**DOI:** 10.1177/02692155231210969

**Published:** 2023-11-09

**Authors:** Johnny Collett, Sophie Lawrie, Sally Bromley, Peter Harling, Alex Reed, Natasha Brusco, Shelly Coe, Jan Coebergh, Camille Carroll, Helen C Roberts, Michele T Hu, Helen Dawes

**Affiliations:** 1 Centre for Movement, Occupational and Rehabilitation Sciences, OxINMAHR 6395 Oxford Brookes University, Oxfordshire, UK; 2 Parkinson's UK Oxford Branch, Oxfordshire, UK; 3 European Parkinson's Therapy Centre, Brescia, Italy; 4 School of Primary and Allied Health Care, Rehabilitation, Ageing and Independent Living (RAIL) Research Centre, Monash University, Frankston, Australia; 5 Centre for Nutrition and Health, OxINMAHR Oxford Brookes University, Oxfordshire, UK; 6 Department of Neurology, St George's University Hospitals NHS Foundation Trust, London, UK; 7 Department of Neurology, Ashford St Peter's NHS Foundation Trust, Chertsey, UK; 8 Translational and Clinical Research Institute, 5994 Newcastle University, Newcastle, UK; 9 Academic Geriatric Medicine, Faculty of Medicine, University of Southampton, Hampshire, UK; 10 Oxford Parkinson's Disease Centre, Division of Neurology, Nuffield Department of Clinical Neurosciences, 6396 University of Oxford, Oxfordshire, UK; 11 NIHR Exeter BRC, Medical School, University of Exeter, Devon, UK

**Keywords:** Carers, Parkinson’s disease, peer support, physical activity, self-management

## Abstract

**Objective:**

A diagnosis of Parkinson's often leads to uncertainty about the future and loss of perceived control. Peer support may offer a means to address these concerns and promote self-management.

**Design:**

A programme evaluation of the feasibility and potential effects of ‘First Steps’, utilising a pragmatic step wedge approach. Comparing First Steps (intervention) to (control) conditions.

**Setting:** In the community at four sites in southern England.

**Participants:** Newly diagnosed (≤ 12months) people with Parkinson's.

**Intervention:** First Steps was a 2-day peer-conceived, developed and led intervention to support self-management.

**Main measures:** At 0, 12 and 24 weeks anxiety and depression (Hospital, Anxiety and Depression Scale, HADS), daily functioning (World Health Organisation Disability Assessment Schedule, WHODAS), physical activity, quality of life (EQ5D), carer strain and service utilisation were assessed.

**Results:**

Between February 2018 and July 2019, 36 participants were enrolled into intervention and 21 to control conditions, all were included in statistical analysis. Lost to follow up was n = 1 (intervention) and n = 1 adverse event was reported (control, unrelated). Of the 36 allocated to the intervention n = 22 participants completed both days of First Steps during the study period. Completion of outcome measures was >95% at 24 weeks. Small effects favouring the intervention were found for HADS (odds ratio (OR) = 2.06, 95% confidence interval (CI) 0.24:17.84), Carer Strain Index (OR = 2.22, 95% CI 0.5:9.76) and vigorous (*d *= 0.42, 95% CI −0.12:0.97) and total physical activity (*d *= 0.41, 95% CI −0.13:0.95). EQ5D, WHOSDAS and service utilisation, was similar between groups.

**Conclusions:**

First Steps was feasible and safe and we found potential to benefit physical activity, mental health and carer strain. Further research with longer-term follow up is warranted.

## Introduction

Parkinson's has no cure and the incidence is increasing; by 2040 a projected 17 million people could be living with the condition worldwide.^1^ In the UK an estimated 70 per 100,000 people are diagnosed every year.^2^ During the diagnosis consultation time is limited and the focus is often on the causes and symptoms of Parkinson's rather than supporting wellbeing.^3^ It is recognised that there is an urgent need to improve the information and support provided, particularly around the time of diagnosis.^4^

A diagnosis of Parkinson's presents psycho-social challenges and adjustments including a loss of perceived control and uncertainty about life.^5^ Knowledge about the illness, symptoms and available treatments can improve a person's sense of control and enable them to make informed decisions about their health management. As such, support for self-management has been recommended as part of Parkinson's rehabilitation programmes.^6^ However, self-management interventions have been found to vary in structure, components and targeted outcomes^6^ resulting in limited evidence for their clinical effectiveness in Parkinson's.^7^ Never-the-less important components of interventions identified include medication management, physical exercise, self-monitoring, psychological strategies, maintaining independence, social engagement, and knowledge and information.^7,8^

Peer support has also been found to produce psycho-social benefits, through sharing experiences, feelings of empowerment and social connectedness, all of which can help people with Parkinson's (PwP) develop new coping skills.^9^ Peer-led interventions, by their nature, benefit from the credibility of the lived experience^10^ and have been promoted as a cost-effective therapeutic opportunity to support others experiencing similar life circumstances.^11^ While being peer led has the potential to add value to self-management interventions few studies have investigated this approach in Parkinson's.^6^ Furthermore informal Parkinson's management and care responsibility often falls on spouses and extended family members of PwP affecting their wellbeing and quality of life.^12^ Specifically considering carers, Parkinson's self-management interventions have been suggested to produce benefits to the carer and the relationship.^13^

To our knowledge a peer-led self-management intervention for newly diagnosed PwP and their carers has not been formally evaluated. Here we aimed to report on a peer-conceived, developed and led intervention to support self-management after a diagnosis of Parkinson's (First Steps). The First Steps programme was consistent with the proposed core components of self-management interventions,^8,14^ and designed also to support carers. It focused on empowering people to face the future positively, and was designed with the intention of national adoption by Parkinson's UK, who funded this evaluation.

## Methods

### Design

A programme evaluation of the feasibility and potential effects of a novel peer-led programme to support self-management for people newly diagnosed with Parkinson's. Initially the study was designed to be a randomised controlled trail. However, following steering group advice the design was amended to use a pragmatic step wedge approach to accommodate the ‘roll out’ of First Steps. Step wedge designs use sequential transition of clusters (sites), from control to intervention conditions.^15^ The evaluation occurred during the first stages of the programme's implementation. Therefore, we used Parkinson's UK roll out schedule for the sequence transition of sites. The evaluation received ethical approval (NRES: 17/SC/0346 and was registered on ISRCTN: 14760402 (https://doi.org/10.1186/ISRCTN14760402).

### Setting and participants

Participants were recruited from Oxfordshire (Oxford University Hospitals NHS Foundation Trust), Hampshire (University Hospital Southampton NHS Foundation Trust), Devon (University Hospitals Plymouth NHS Trust) and Surrey (Ashford and St Peter's Hospitals NHS Foundation Trust) participant identification centres (PIC). The First Steps programme was delivered in local community facilities.

We aimed to recruit a convenience sample of 80 participants, n = 40 to received First Steps and n = 40 to control conditions (usual care), when First Steps was not available at their location. Clinicians at PICs screened clinic lists for potentially eligible patients and informed them about the research and provided the participant information. Consent was obtained for those interested to be contacted a member of the research team. To be included in the research participants needed: (1) a diagnosis of Parkinson's disease within 12 months and (2) be 18 years or older. Participation was excluded if individuals had: (1) severe depression or psychosis, (2) reduced cognition that would preclude active involvement and capacity to consent to participate or (3) unable to understand English.

### First steps

**Figure 1.**
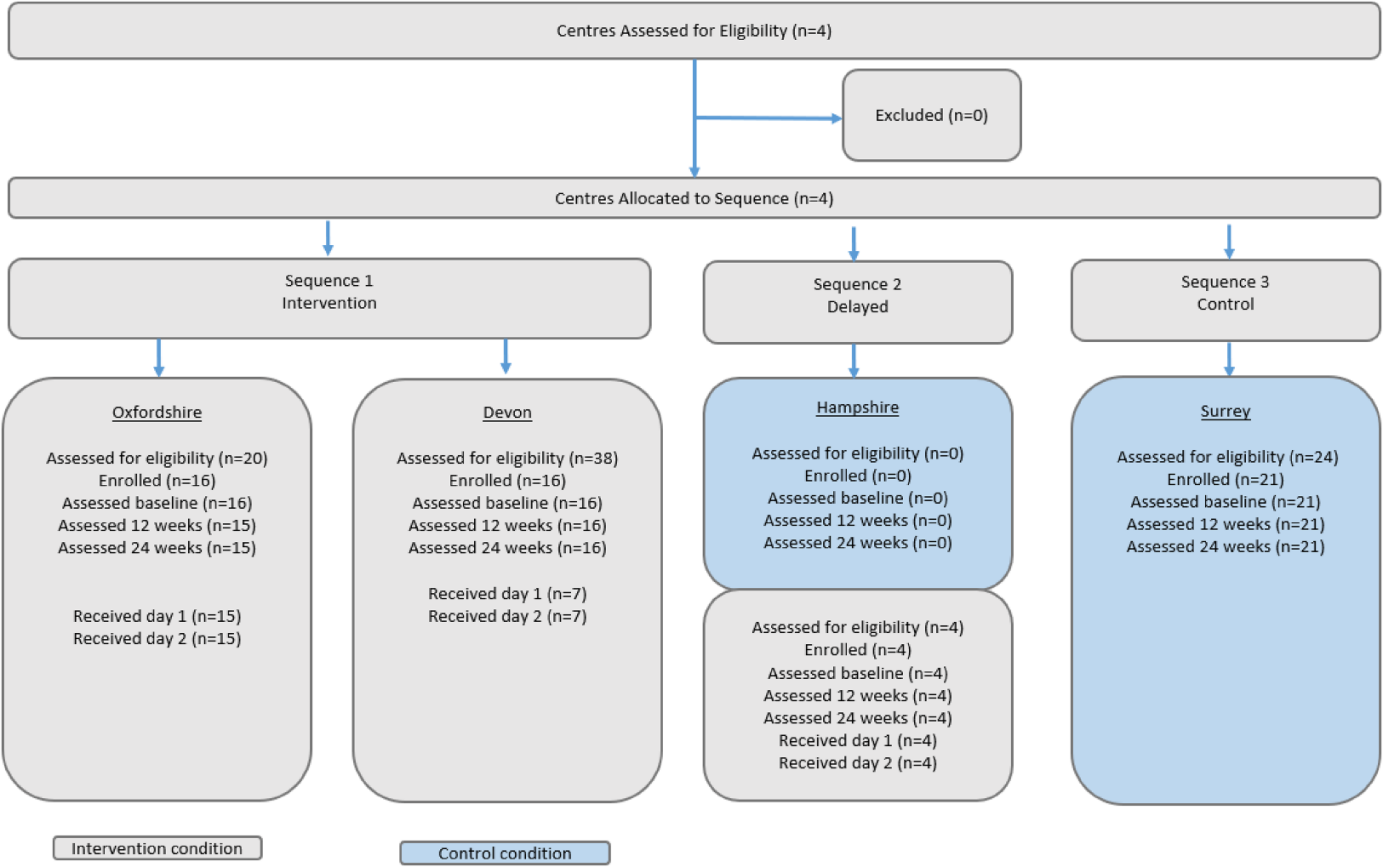
Study flow.

Programme delivery: Three centres were pragmatically allocated to implement First Steps starting at successive time points, according to the ‘roll out’ schedule determined by Parkinson's UK. First Steps was not scheduled to be rolled out to one of the participating centres (Surrey) during the study time period. Figure 1 provides an overview.

Programme Development: First Steps was conceived and developed by PwP (PH, SB and AR). Development of the intervention content was iterative with consultation with experts. A workshop was held with stakeholders in June 2015 to finalise content (Supplement 1). First Steps was initially piloted in Oxfordshire prior to this programme evaluation. A steering group was established that had oversight of the programme content and documentation and approved requests for changes. First Steps is briefly described below considering the *template for intervention description and replication (TIDieR*),^16^ with more details available in Supplement 2.

First Steps provided information and facilitated discussion with the objective to empower people to take control of their Parkinson's early after diagnosis through peer-facilitated sessions. Sessions were delivered on 2 separate days within a 6-week period. Each day started at 9 am and ended at 2 pm with a lunch break.

Day 1 covered: (1) Parkinson's and medication, (2) the impact of diagnosis and how to face the future positively, (3) addressing fears and misconceptions around the condition, (4) accessing the right services/specialists, (5) getting the right information and support, and (6) the importance of exercise and lifestyle in managing the condition.

Day 2 covered: (1) a review of how participants have been getting on since day 1, such as activities they have taken up and a discussion on successes/challenges, (2) rights in relation to Parkinson's (e.g. employment, driving, prescriptions, changing doctors), (3) practical facts related to Parkinson's, (3) separate group discussions on how loved ones can best support their partner or relative and be supported themselves, and (4) a ‘taster’ exercise session and information on local exercise classes. Attendance was free and free parking and lunch was available.

The First Steps programme was delivered by facilitators recruited by Parkinson's UK. Facilitators were PwP from the locality where First Steps was being delivered. Facilitators were trained by experienced facilitators (from Oxfordshire) and Parkinson's UK. Facilitators were expected to deliver the materials provided according to the programme protocol (Supplement 2). Facilitators were observed delivering at least two sessions to assess adherence to content and principles. The exercise taster session was provided by a local neuro physiotherapist. Delivery was via face-to-face group sessions of up to 6 PwP accompanied by partners and/or other supportive persons (up to 12 people total). Sessions were facilitator led and supported by slides and video content. Sessions were designed to facilitate discussion and be interactive.

First Steps took place in a non-clinical setting (i.e. hotel with leisure facilities). Criteria for delivery centres were (1) equipped meeting rooms appropriate to run the seminars and support functions, (2) easy access and free parking, (3) catering available, and (4) access to exercise equipment. While the content and materials of the First Steps programme were standardised, sessions where interactive and a core component was that individual circumstances and concerns were discussed.

### Programme evaluation

Group allocation was according to location, participants would be aware if they were offered First Steps or not. Assessments were over the telephone with an assessor blind to participants’ location (a paper version was available on request – sent by non-blind researcher). Participant's telephone numbers were entered into the designated assessment telephone by an unblind researcher so that area code was not displayed and participants were reminded not to disclose their location at the start of each assessment call.

Demographic information was ascertained at entry to the study (baseline assessment). Given the pragmatic approach to site allocation and the potential for imbalance post code was used to classify deprivations indices (Ministry of Housing Communities and Local government look up tool, 2019) and location (Rural-Urban Classification for Output Areas, 2011 Office for National statistics). Outcome data was obtained at 0 (baseline), 12 and 24 weeks after entry to the study by the same assessor blind to location. After a minimum of three missed call attempts and no reply to messages a participant was deemed lost to follow up.

Here we report outcomes of *Anxiety and depression:* Hospital, Anxiety and Depression Scale (HADS), 14 items (7 anxiety and 7 depression) on a 4-point scale from 0 (absent) to 3 (extreme); *Daily functioning:* World Health Organisation Disability Assessment Schedule (WHODAS) 12 items across 6 domains, total score 0 (no disability) −100, (full disability); *Physical activity*: International Physical Activity Questionnaire-short (IPAQ), amounts of weekly walking, moderate, vigorous physical activity estimated in metabolic equivalents (METs). *General health/quality of life*: EQ5D-5L, 5 domains (mobility, self-care, usual activities, pain/discomfort and anxiety/depression) rated on 5 levels from 1 (no problems) to 5 (extreme/unable) and a 0 (worst health) to 100 (best health) index. *Healthcare utilisation*: Modified Client Service receipt inventory of healthcare services used. Additionally, supportive persons were asked to complete the Carer Strain Index (CSI), sum of 12 yes/no items.

Adherence to the intervention was measured as attendance at First Steps. Participant experience is reported in detail elsewhere.^17^ Fidelity of intervention delivery was assessed through the observation of sessions at an intervention centre by a member of the research team using check list for programme quality, venue and facilitator (Supplement 3 for check lists).

### Analysis

Sample size: This was a programme evaluation to determine feasibility and potential effects. We predicted anxiety would be high after diagnosis and 40 people per group would enable us to detect large between-group responses in anxiety (2.5-point difference with standard deviation as large as 3, with 80% power to detect a large effect). This sample size was also deemed sufficient to describe recruitment, retention, adherence, completion of measures and estimate variability in outcomes (to inform future sample size calculations).

Statistical analysis followed the intention-to-treat principle in that data from all individuals enrolled were included in analyses according to group. Analysis was between-group and pre–post intervention. The 12-week assessment was intended as the post-intervention point. However, n = 11 individuals had not completed both days by 12 weeks and their 24-week assessment data was used for post-intervention. The linear mixed models (LMM) procedure of SPSS V.28 was used to determine change in continuous variables, reported with Cohen’s d and 95% confidence intervals (CI). The model utilised maximum likelihood estimation and included individual intercept as a random effect. Ordinal measures were analysed using the generalised mixed model procedure of SPSS reported with odds ratios and 95% CIs. Odds ratios of 1.68, 3.47 and 6.71 and Cohen's d of 0.2, 0.5 and 0.8 where interpreted as small, medium and large, respectively.^18^ Healthcare utilisation analysis reported the percentage of PwP who utilised various healthcare resources over a 12-month time horizon. This is based on 6 months prior to the intervention commencing (First Steps or usual care), and in the 6 months following the intervention commencement (First Steps or usual care) via the 24-week assessment, *X*^2^ was used to compare groups. Alpha was p = 0.05.

## Results

### Participants

Participant flow can be found in Figure 1, between February 2018 and July 2019, 57 out of 86 PwP assessed for eligibility were enrolled (Oxfordshire n = 16, Hampshire n = 4, Devon n = 16 and Surrey n = 21), n = 36 to intervention and n = 21 to control conditions. Thus, we did not recruit to target within the study period. Reasons for not enrolling on the study were: n = 3 (intervention) were over 1 year since diagnosis, n = 2 (intervention) had already been on the First Steps pilot, n = 2 specifically responded they were not interested in taking part (n = 1 intervention, n = 1 control), n = 1 stated they did not have the time (intervention), n = 21 did not give reasons.

Attrition was minimal, 1 individual withdrew without giving reason and was lost to follow up (Intervention). There was one adverse event reported, a fall resulting in hospitalisation (control group). The fall was deemed unrelated and the participant completed the study. Completion of outcome measures was high (>95% at 24 weeks), median number of call attempts was 2 (IQR 2–4). The carer giver strain index was completed by n = 43 (n = 26 intervention, n = 17 control).

Demographic data is shown in Table 1, there was no difference in age or gender between groups. Location classification differed between groups (p≤0.001). Regardless of group most people lived in urban locations, the control all lived in urban locations and the majority in a major conurbation (p≤0.05). Those in the control group also lived in areas with significantly less deprivation (multiple indices: p = 0.003, income effecting older people: p = 0.012). There was no difference between groups for outcomes at baseline except for EQ5D usual activities, with those in the intervention group reporting greater problems (p = 0.035). While there was no statistical difference between groups in physical activity, the control group tended to be more active (total METs p = 0.151) especially in the walking domain (p = 0.096). HADS total (p = 0.201) and depression (p = 0.192) scores also trended towards being higher in the intervention group (Table 2).

**Table 1.** Demographic data.

	First Steps	Control	Between group
*Age*			
Years	*68 ± 8*	68 *± 8*	p = 0.554
>65	*22* (*61.1%)*	14 (66.7%)	p = 0.675
*Gender*			
Male: female	*22:14*	14:7	p = 0.675
*Rural-Urban Classification*			*X*^2 ^= 42.56, p≤0.001
Rural hamlets	6 (16.7)	0 (0.0%)	p≤0.05
Rural town and fringe	4 (11.1%)	0 (0.0%)	NS
Rural village	5 (13.9%)	0 (0.0%)	NS
Urban city and town	21 (58.3%)	4 (19.0%)	p≤0.05
Urban major conurbation	0 (0.0%)	17 (81.0%)	p≤0.05
Deprivation indices			
Index of deprivation	7 (5–10)	10 (8.5–10)	p = 0.003
Health and disability decile	8 (5–10)	10 (8.5–10)	p = 0.107
IDAOPI decile	7 (6–9)	10 (.7.5–10)	p = 0.012

Descriptive statistic report mean ± standard deviation, median (interquartile range) or *n* (%). IDAOPI: Income Deprivation Affecting Older People Index; NS: non-significant; *p*: probability value from between-group analysis using independent samples t test, Mann-Whitney U or Persons Chi Squared.

**Table 2.** Outcomes.

	First Steps										Control										
	Pre (n = 36)					Post (n = 35)					Pre (n = 21)					Post (n = 21)					Between group
*HADS*	Median (IQR)					Median (IQR)					Median (IQR)					Median (IQR)					OR (95% CI), p
Anxiety	*3.5 (2–5)*					3 (1–6)					*2 (0–4.5)*					2 (0.5–5)					1.61 (0.39:6.57), *0.507*
Depression	*3 (1–5)*					2 (1–4)					*1 (1–3)*					2 (1–4)					1.28 (0.42:3.92), *0.664*
Total	*6 (3–10)*					4 (−3–10)					*4 (1–9)*					4 (2.5–8)					2.06 (0.24:17.84), *0.511*
*WHODAS*	Median (IQR)					Median (IQR)					Median (IQR)					Median (IQR)					OR (95% CI), p
Total	*15 (13–18)*					15 (12–20)					16 (13.5–19)					15 (12.5–18.5)					1.58 (0.19:13.3), *0.674*
*IPAQ*	EMM ± standard error					EMM ± standard error					EMM ± standard error					EMM ± standard error					d (95% CI), p
Walking (METs)	547 ± 93					659 ± 94					767 ± 123					880 ± 121					0.38 (−0.16:0.92), *0.132*
Moderate (METs)	408 ± 60					360 ± 61					477 ± 81					397 ± 79					0.19 (−0.35:0.73), *0.688*
Vigorous (METs)	422 ± 111					731 ± 114					395 ± 153					318 ± 146					0.42 (−0.12:0.97), *0.075*
Total (METs)	1340 ± 153					1776 ± 156					1631 ± 206					1595 ± 198					0.41 (−0.13:0.95), *0.092*
Carer Strain Index	Median (IQR)					Median (IQR)					Median (IQR)					Median (IQR)					OR (95% CI), p
Total	1.5 (0–4), n = 26					1 (0–3), n = 26					1 (0–5), n = 16					1 (0–2.5), n = 17					2.22 (0.5:9.76), *0.290*
EQ5D-5L	Median (IQR)					Median (IQR)					Median (IQR)					Median (IQR)					OR (95% CI), p
VAS	80 (70–88.75)					80 (70–90)					90 (80–95)					90 (75–92.5)					0.01 (0.00:13.32), 0.180
Domains	1	2	3	4	5	1	2	3	4	5	1	2	3	4	5	1	2	3	4	5	
Mobility	21	14	1	0	0	21	11	3	0	0	10	7	3	1	0	9	10	1	0	1	0.76 (0.48:1.19), *0.228*
Self-care	30	5	1	0	0	30	5	0	0	0	18	3	0	0	0	16	5	0	0	0	0.91 (0.73:1.13), 0.387
Usual activity	18	15	3	0	0	19	15	1	0	0	13	7	1	0	0	18	1	1	1	0	1.22 (0.84:1.78), 0.296
Pain	10	16	7	3	0	14	17	4	0	0	9	9	0	0	0	11	5	4	1	0	0.95 (0.61:1.47), 0.836
Anxiety depression	19	13	4	0	0	19	13	3	0	0	13	8	0	0	0	13	6	1	1	0	1.0 (0.68:1.52), 0.926

Descriptive statistics: EMM (Estimated Marginal Means) ± standard error estimates, median (interquartile range) or n. Inferential statistics: d = Cohen's d with (95% confidence intervals (CI)). EQ5D: Euro-QOL EQ5D-5L; HADS: Hospital, Anxiety and Depression Scale; IPAQ: International Physical Activity Questionnaire-short; MET: metabolic equivalents; OR: odds ratio with (95% CI); Pre: intervention assessment point; Post: post intervention assessment point; VAS: visual analogue scale; WHODAS: World Health Organisation Disability Assessment Schedule.

### Programme fidelity

Figure 1 shows n = 22 of the 36 PwP completed both days of the intervention during the study period, all those who completed day 1 also completed day 2. Eight people did not start the intervention and gave no reason, n = 1 was scheduled but did not attend and n = 1 remained on the intervention waiting list for the duration of the study period. Participants experience of the intervention was positive and reported in detail elsewhere.^17^

Fidelity of delivery was assessed at Oxfordshire (April 2018 – day 2) and Devon (December 2018 – day 1). Fidelity was not assessed at the Hampshire site as the delivery of the programme was delayed due to recruitment and training of facilitators. The only issue on the programme quality check list at the Oxfordshire centre was not having both male and female facilitators (the 2 facilitators were men). The Devon centre also failed this item (the 2 facilitators were women), and also altered content on mental health. Specifically, on day 1 a presentation slide had been removed and group discussion around anxiety and stress did not occur. Venue and facilitator required no action at Oxfordshire and Devon centres.

### Outcome

Outcome data are reported in Table 2, no significant differences were found between groups. Small effect sizes were found for HADS total, CSI, and vigorous and total physical activity favouring those attending programme. Within group pre–post differences were significant in the intervention group for total (p = 0.023, d = 0.56 (95% CI = 0.08:1.05)) and vigorous (p = 0.046, d = 0.49 (95% CI = 0.01:0.965)) activity.

Healthcare resource utilisation was similar between groups over the 12-month time horizon, and within groups between the two 6-month periods (Supplement 4). A high portion of the participants had a hospital-based neurological outpatient appointment, a neurologist appointment, a general practitioner appointment and a nurse appointment, in one or both 6-month periods. A greater portion of participants in the First Steps programme had contact with a physiotherapist (both time periods) and nurse (latter time period only), and a lesser portion having an magnetic resonance imaging (former time period only). Estimated per person cost of delivering the intervention ranged between £150 and £630 (depending on number of persons attending, venue hire and lunch, facilitator expenses and physiotherapist costs). This cost does not include costs incurred by the individual such as travel or missing work (PwP and/or supportive person).

## Discussion

We found First Steps, conceived, developed and delivered by PwP, administered by a national charity, and with newly diagnosed patients identified by National Health services, was successfully delivered at different sites and largely maintained good fidelity. We found potential benefit to mental health and carer strain, and the results for increasing physical activity were particularly encouraging.

Our results of increased physical activity in the intervention group are consistent with insights from our qualitative study that indicated First Steps improved physical activity behaviour.^17^ Promoting physical activity and exercise in those with Parkinson's is recommended,^19^ especially for those in the early stages of the condition.^20^ Previously in a trial of community-based exercise we found excellent adherence to a largely self-managed 6-month exercise programme that led to improvement in motor symptoms.^21^ There is an increasing amount of evidence that habitual exercise can delay the clinical course of Parkinson's.^22,23^ Supported by animal models that have identified mechanisms of how exercise may affect the pathophysiology of Parkinson’s.^24^ Furthermore, a recent trial in those at the early stages of Parkinson's indicated that high intensity exercise may elicit greater benefits to motor symptoms.^25^ Therefore, the increase in vigorous physical activity levels we found is particularly encouraging. These results are consistent with findings of our qualitative study in which participants reported they had increased level, intensity or type of physical activity following First Steps.^17^ Whilst, we cannot infer long-term behaviour change, these results combined demonstrate the potential and importance of interventions to support physical activity behaviour in those newly diagnosed. It should be noted that both groups were physically active at baseline, particularly the control group, with average PA levels consistent with those that accrue health benefits.^26^ Indeed, the population in the current study were from areas of low deprivation which are associated with healthier lifestyles.^27^ It is also of note that the exercise component included a practical session with a health professional, expert advice and reassurance been found to reduce barriers to participating in community exercise.^21,28^

Anxiety and depression are common in de novo Parkinson's^29^ and we assumed anxiety would be high in this population when estimating sample size. However, anxiety and depression were not prevalent in our sample. The evidence for psychological intervention to improve anxiety in Parkinson's has been found to be inconsistent.^30^ Simons et al.^31^ investigated an education programme also aiming to empower PwP, they found immediate improvement in mood but no significant changes in depression. While we observed a small effect in total HADS score, follow-up time may not have been long enough for effects to be fully realised as benefits may appear later as participants encounter challenges in life.^31^ Indeed, our qualitative^17^ study found, the psychological and social mechanisms that may explained the impact of First Steps, were perceived control, hope and action, the individual's mind-set, and perceived confidence. Outcomes centred in these constructs are consistent with the aim of the intervention and may have been more appropriate over the time period of the current study. Indeed, self-efficacy has been found to be a predominant factor in promoting self-management in Parkinson's.^32^ The use of peers has been advocated as a means to support self-efficacy^33^ and peer-led self-management intervention have been promoted for their cost-effective potential.^9,11^ Our findings that, with training, intervention fidelity was largely maintained across sites delivered by different peer facilitators is therefore promising. Involving careers was a central component of First Steps and improved self-management has been associated with informal support from ‘carers’.^34^ Furthermore, we found potential effect on carer strain which is also encouraging. Lyons et al.^13^ found declines in depressive symptoms for spouses, after a couples self-management programme for Parkinson's. Their community peer support programme also found both PwP and spouses reported positive self-management behaviours including increased weekly aerobic physical activity.^13^

When evaluating the result of this study it should be considered that originally the design was intended to be a feasibility randomised controlled trial. While, the necessitated change, to a more pragmatic evaluation had ecological advantages, it also created challenges. Firstly, attendance at First Steps did not align with the assessment schedule in all individuals. This meant the intended follow-up analysis was not possible as the final assessment was the post-intervention assessment in approximately a third of individuals. In addition, one site was delayed and had difficulties recruiting and training facilitators, affecting the control sequence of the step wedge design and the study recruiting to target during the study period. While we found good intervention fidelity, we did observe a change to important programme content, thus monitoring of sites would be indicated in further roll out. The proportion of people enrolled from those identified was less in Devon which is more rural than Oxfordshire with potentially greater time and cost for travel to the First Steps delivery site. Furthermore, it should be considered that the evaluation took place in relativity affluent locations and the potential selection bias in our results, with participants being largely active and exhibiting only mild anxiety or depression. Indeed, the current study would have benefited for including measures of control and coping. Future studies should consider including these outcomes as well as engagement strategies to reach those who may be experiencing worse mental health. The demographic across sites was a largely a retirement aged population. Whilst, this is indicative of the age at which most people are diagnosed^1^ tailoring may be required for those diagnosed at younger. A recent survey of those in work at diagnosis, reported that only 22% received an early intervention to support self-management and that symptoms and required support may differ in those diagnosed younger.^35^ It is fundamental to the context of this report that the evaluation took place before the COVID-19 pandemic. During the pandemic First Steps moved to online delivery, which has endured to date. While the benefits of peer support are not limited to the in person setting and online delivery has advantages of being more widely accessible, not requiring travel and easier to standardise,^9^ we would be cautious to directly translate the current results to the online delivery.

Self-management interventions have been advocated as a potential low-cost way to reduce hospital use and total costs for chronic conditions^11^ as well as accrue benefits to the individual and those who support them.^13^ We found potential for effect, especially for improving physical activity, and no indication of harm associated with the programme. The study provides data that it is feasible to establish peer-led self-management to support those newly diagnosed with Parkinson's. Future research is required to establish efficacy and generalisability to a broader demographic. We would recommend a long follow-up period to determine if individual and economic benefits are realised and further implementation should ensure it is reaching all those who may benefit.

**Clinical messages**

- It was feasible to implement, a peer-conceived, developed and delivered self-management intervention for people newly diagnosed with Parkinson's (First Steps).
- First Steps was safe and could potentially improve physical activity, especially through participation in more vigorous intensity exercise.
- Further implementation of First Steps should evaluate and ensure it is reaching all those who may benefit.

## Supplemental Material

sj-docx-1-cre-10.1177_02692155231210969 - Supplemental material for A programme evaluation of ‘First Steps’: A peer-conceived, developed and led self-management intervention for people after a Parkinson's diagnosis Supplemental material, sj-docx-1-cre-10.1177_02692155231210969 for A programme evaluation of ‘First Steps’: A peer-conceived, developed and led self-management intervention for people after a Parkinson's diagnosis by Johnny Collett, Sophie Lawrie, Sally Bromley, Peter Harling, Alex Reed, Natasha Brusco, Shelly Coe, Jan Coebergh, Camille Carroll, Helen C Roberts, Michele T Hu and Helen Dawes in Clinical Rehabilitation

## References

[1] Dorsey ER Sherer T Okun MS , et al. The emerging evidence of the Parkinson pandemic. J Parkinsons Dis 2018; 8: S3–S8. 10.3233/JPD-181474 PMC6311367 30584159

[2] Okunoye O Marston L Walters K , et al. Change in the incidence of Parkinson's disease in a large UK primary care database. NPJ Parkinsons Dis 2022; 8: 23. 35292689 10.1038/s41531-022-00284-0 PMC8924194

[3] Schrag A Modi S Hotham S , et al. Patient experiences of receiving a diagnosis of Parkinson's disease. J Neurol 2018; 265: 1151–1157. 29546451 10.1007/s00415-018-8817-8 PMC5937885

[4] Alliance TN . Falling Short has the neurology patient experience changed since 2014. 2017.

[5] Soundy A Stubbs B Roskell C . The experience of Parkinson's disease: a systematic review and meta-ethnography. Sci World J 2014; 2014: 613592. 10.1155/2014/613592 PMC4265687 25525623

[6] Kessler D Liddy C . Self-management support programs for persons with Parkinson's disease: an integrative review. Patient Educ Couns 2017; 100: 1787–1795. 28465112 10.1016/j.pec.2017.04.011

[7] Pigott JS Kane EJ Ambler G , et al. Systematic review and meta-analysis of clinical effectiveness of self-management interventions in Parkinson's disease. BMC Geriatr 2022; 22: 45. 35016613 10.1186/s12877-021-02656-2 PMC8753859

[8] Armstrong M Tuijt R Read J , et al. Health care professionals’ perspectives on self-management for people with Parkinson's: qualitative findings from a UK study. BMC Geriatr 2021; 21: 706. 34911497 10.1186/s12877-021-02678-w PMC8672490

[9] Gerritzen EV Lee AR McDermott O , et al. Online peer support for people with Parkinson disease: narrative synthesis systematic review. JMIR Aging 2022; 5: e35425. 10.2196/35425 PMC9377481 35896025

[10] Barclay L Hilton GM . A scoping review of peer-led interventions following spinal cord injury. Spinal Cord 2019; 57: 626–635. 31123333 10.1038/s41393-019-0297-x

[11] Aterman S Ghahari S Kessler D . Characteristics of peer-based interventions for individuals with neurological conditions: a scoping review. Disabil Rehabil 2023; 45: 344–375. 35085058 10.1080/09638288.2022.2028911

[12] Gumber A Ramaswamy B Thongchundee O . Effects of Parkinson's on employment, cost of care, and quality of life of people with condition and family caregivers in the UK: a systematic literature review. Patient Relat Outcome Meas 2019; 10: 321–333. 31695537 10.2147/PROM.S160843 PMC6816078

[13] Lyons KS Zajack A Greer M , et al. Benefits of a self-management program for the couple living with Parkinson's disease: a pilot study. J Appl Gerontol 2021; 40: 881–889. 32401118 10.1177/0733464820918136

[14] Shah R Read J Davies N , et al. People with Parkinson's perspectives and experiences of self-management: qualitative findings from a UK study. PLoS One 2022; 17: e0273428. 10.1371/journal.pone.0273428 PMC9462566 36083947

[15] Copas AJ Lewis JJ Thompson JA , et al. Designing a stepped wedge trial: three main designs, carry-over effects and randomisation approaches. Trials 2015; 16: 52. 26279154 10.1186/s13063-015-0842-7 PMC4538756

[16] Hoffmann TC Glasziou PP Boutron I , et al. Better reporting of interventions: Template for Intervention Description and Replication (TIDieR) checklist and guide. BMJ (Clin Res ed) 2014; 348: g1687. 10.1136/bmj.g1687 24609605

[17] Soundy A Collett J Lawrie S , et al. A qualitative study on the impact of first steps—a peer-led educational intervention for people newly diagnosed with Parkinson's disease. Behav Sci (Basel) 2019; 9: 107. DOI: 10.3390/bs9100107 31658668 10.3390/bs9100107 PMC6826464

[18] Chen H Cohen P Chen S . How big is a big odds ratio? Interpreting the magnitudes of odds ratios in epidemiological studies. Commun Stat – Simul Comput 2010; 39: 860–864.

[19] Fox SH Katzenschlager R Lim SY , et al. International Parkinson and movement disorder society evidence-based medicine review: update on treatments for the motor symptoms of Parkinson's disease. Mov Disord 2018; 33: 1248–1266. 29570866 10.1002/mds.27372

[20] NICE. Parkinson’s disease in adults. *NICE guideline [NG71]* 2017.

[21] Collett J Franssen M Meaney A , et al. Phase II randomised controlled trial of a 6-month self-managed community exercise programme for people with Parkinson's disease. J Neurol Neurosurg Psychiatry 2017; 88: 204–211. 27837101 10.1136/jnnp-2016-314508

[22] Tsukita K Sakamaki-Tsukita H Takahashi R . Long-term effect of regular physical activity and exercise habits in patients with early Parkinson disease. Neurology 2022; 98: e859–e871. 10.1212/WNL.0000000000013218 PMC8883509 35022304

[23] Sacheli MA Murray DK Vafai N , et al. Habitual exercisers versus sedentary subjects with Parkinson's disease: multimodal PET and fMRI study. Mov Disord 2018; 33: 1945–1950. 30376184 10.1002/mds.27498

[24] Lai JH Chen KY Wu JC , et al. Voluntary exercise delays progressive deterioration of markers of metabolism and behavior in a mouse model of Parkinson's disease. Brain Res 2019; 1720: 146301. 31226324 10.1016/j.brainres.2019.146301 PMC6702069

[25] Schenkman M Moore CG Kohrt WM , et al. Effect of high-intensity treadmill exercise on motor symptoms in patients with de novo Parkinson disease: a phase 2 randomized clinical trial. JAMA Neurol 2018; 75: 219–226. 29228079 10.1001/jamaneurol.2017.3517 PMC5838616

[26] Huang BH Duncan MJ Cistulli PA , et al. Sleep and physical activity in relation to all-cause, cardiovascular disease and cancer mortality risk. Br J Sports Med 2022; 56: 718–724. 34187783 10.1136/bjsports-2021-104046

[27] Foster HME Celis-Morales CA Nicholl BI , et al. The effect of socioeconomic deprivation on the association between an extended measurement of unhealthy lifestyle factors and health outcomes: a prospective analysis of the UK Biobank cohort. Lancet Public Health 2018; 3: e576–e585. 10.1016/S2468-2667(18)30200-7 30467019

[28] Elsworth C Winward C Sackley C , et al. Supported community exercise in people with long-term neurological conditions: a phase II randomized controlled trial. Clin Rehabil 2011; 25: 588–598. 21382866 10.1177/0269215510392076

[29] Yang N Ju Y Ren J , et al. Prevalence and affective correlates of subjective cognitive decline in patients with de novo Parkinson's disease. Acta Neurol Scand 2022; 146: 276–282. 35722712 10.1111/ane.13662 PMC9545461

[30] Zarotti N Eccles FJR Foley JA , et al. Psychological interventions for people with Parkinson's disease in the early 2020s: where do we stand? Psychol Psychotherapy Theory Res Practice 2021; 94: 760–797. 10.1111/papt.12321 33174688

[31] Simons G Thompson SB Pasqualini S , et al. An innovative education programme for people with Parkinson's disease and their carers. Parkinsonism Relat Disord 2006; 12: 478–485. 16781881 10.1016/j.parkreldis.2006.05.003

[32] Chenoweth L Gallagher R Sheriff JN , et al. Factors supporting self-management in Parkinson's disease: implications for nursing practice. Int J Older People Nurs 2008; 3: 187–193. 20925819 10.1111/j.1748-3743.2008.00123.x

[33] Lorig KR Holman H . Self-management education: history, definition, outcomes, and mechanisms. Ann Behav Med 2003; 26: –7. 10.1207/S15324796ABM2601_01 12867348

[34] Chenoweth L Sheriff J McAnally L , et al. Impact of the Parkinson's disease medication protocol program on nurses’ knowledge and management of Parkinson's disease medicines in acute and aged care settings. Nurse Educ Today 2013; 33: 458–464. 22626862 10.1016/j.nedt.2012.04.022

[35] Collett J Brusco N Cordell N , et al. Lost employment potential and supporting people with Parkinson's to stay in work: insights from a Pan European cross-sectional survey. Disabil Rehabil 2023; 45: 832–839. 35249423 10.1080/09638288.2022.2043460

