# Supplementary material for: A programme evaluation of ‘First Steps’: A peer-conceived, developed and led self-management intervention for people after a Parkinson's diagnosis
Source: Clin Rehabil. 2023 Nov 9;38(3):403–13. doi: 10.1177/02692155231210969 (PMC10829422; doi:10.1177/02692155231210969)

Supplementary information

A Programme evaluation of ‘First Steps’: A peer conceived, developed and led self-management intervention for people after a Parkinson’s diagnosis

**Supplement 1**


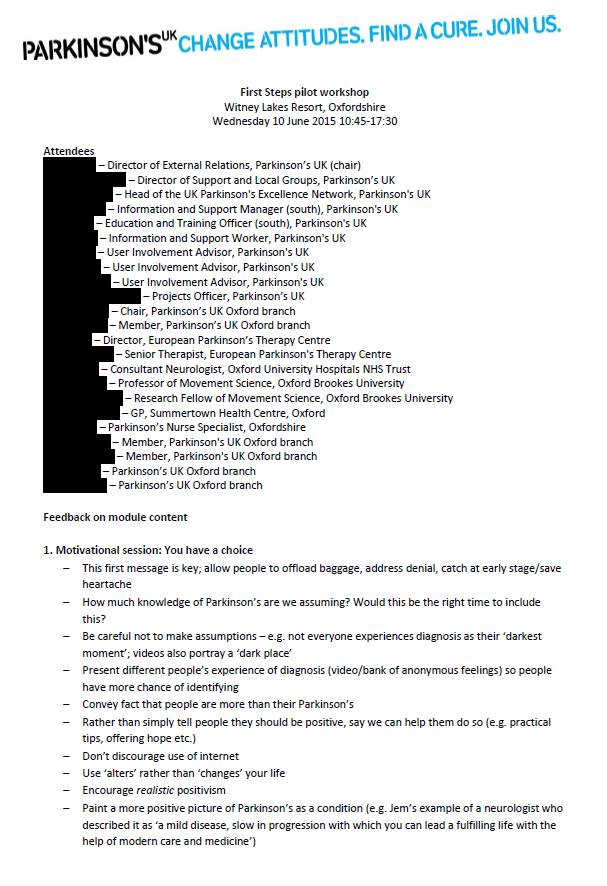


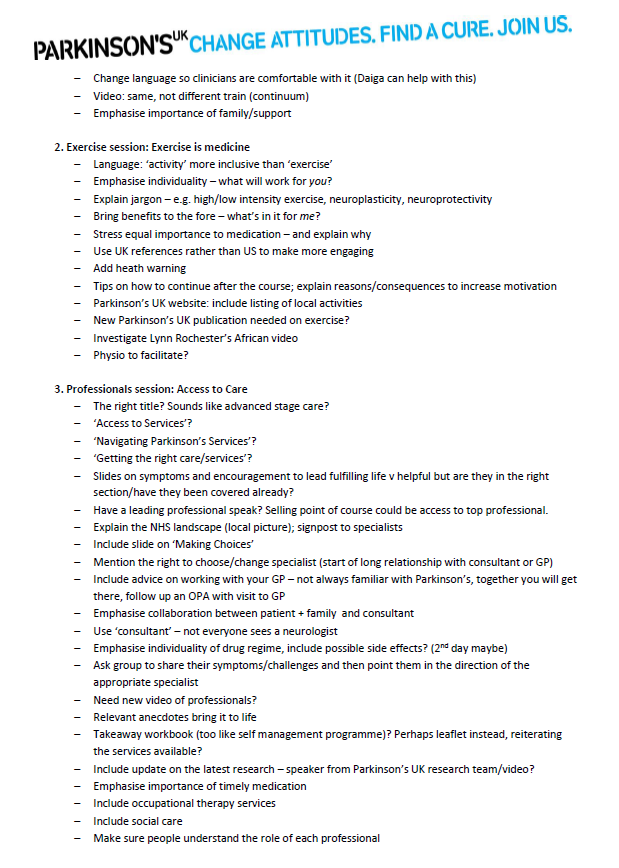


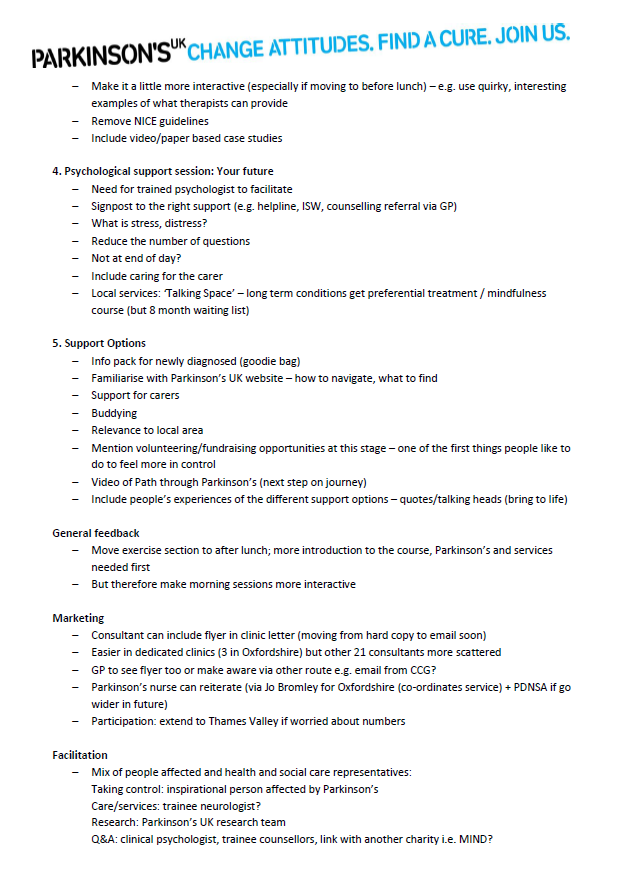


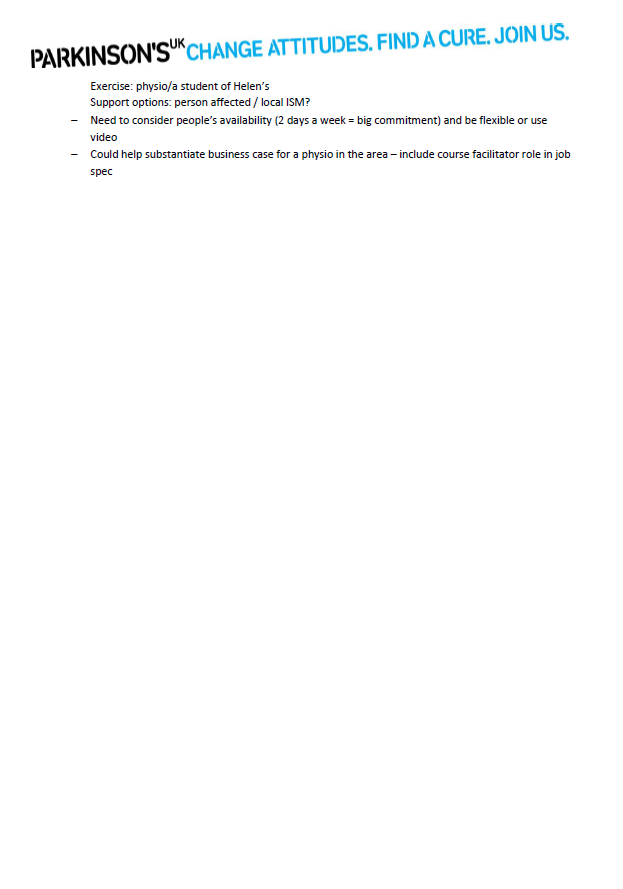


**Supplement 2**


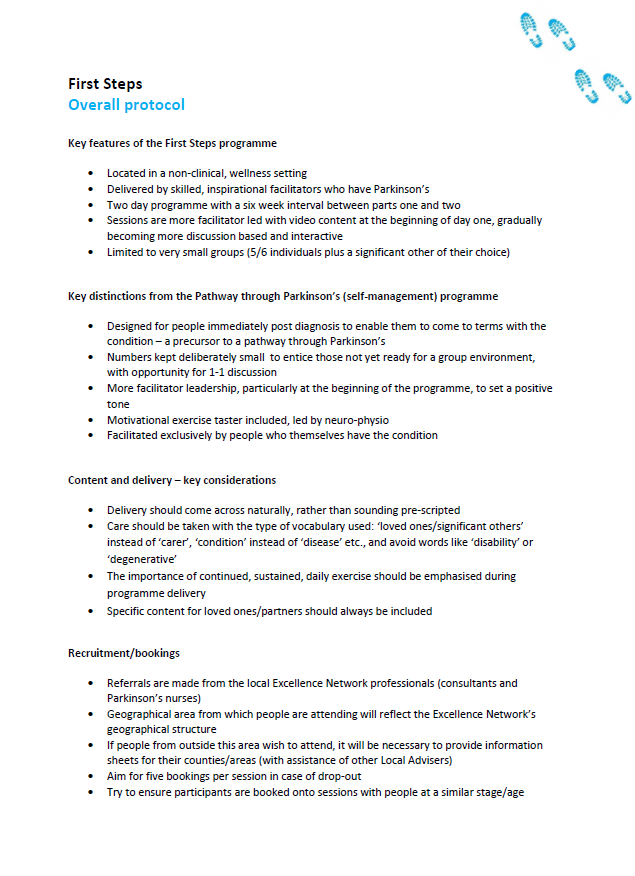


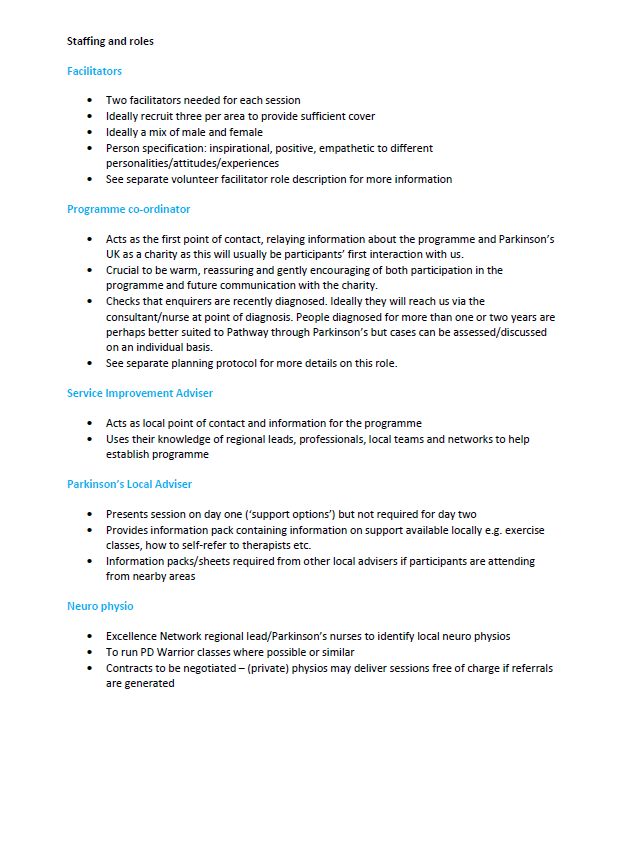


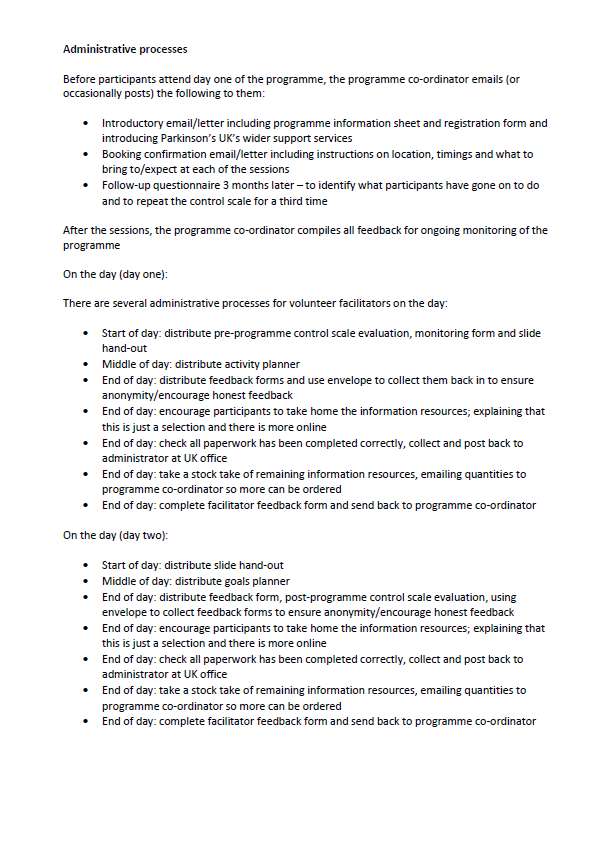


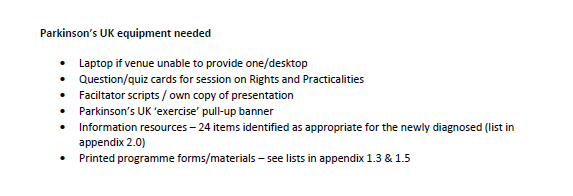

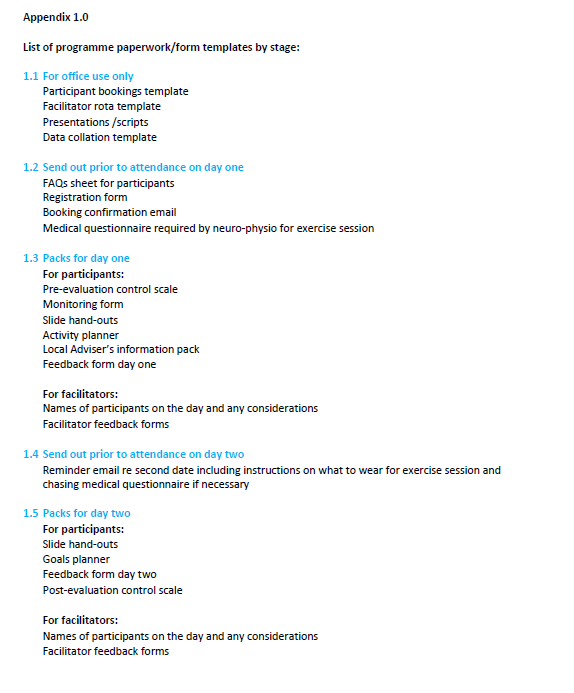


**Supplement 3**


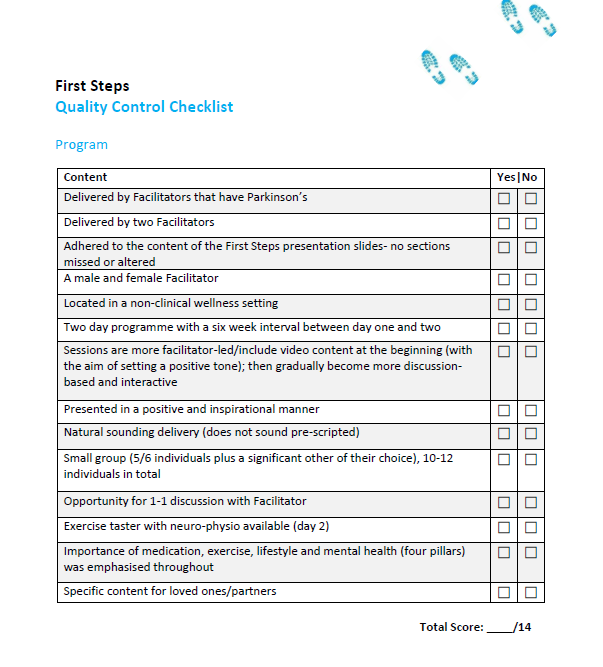


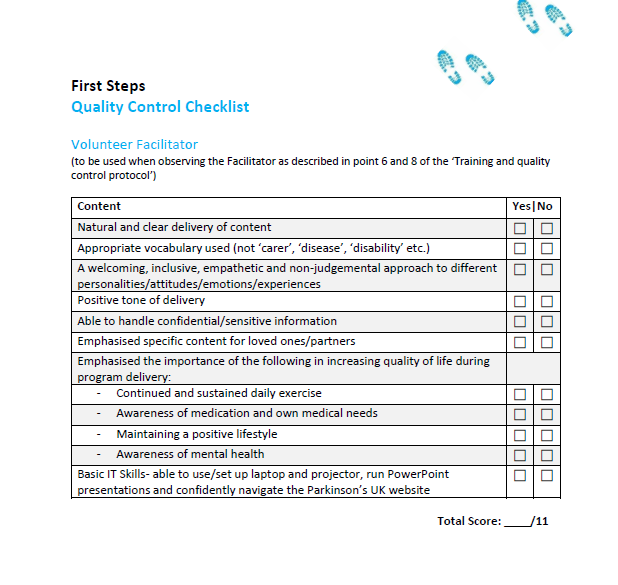


**Supplement 4**


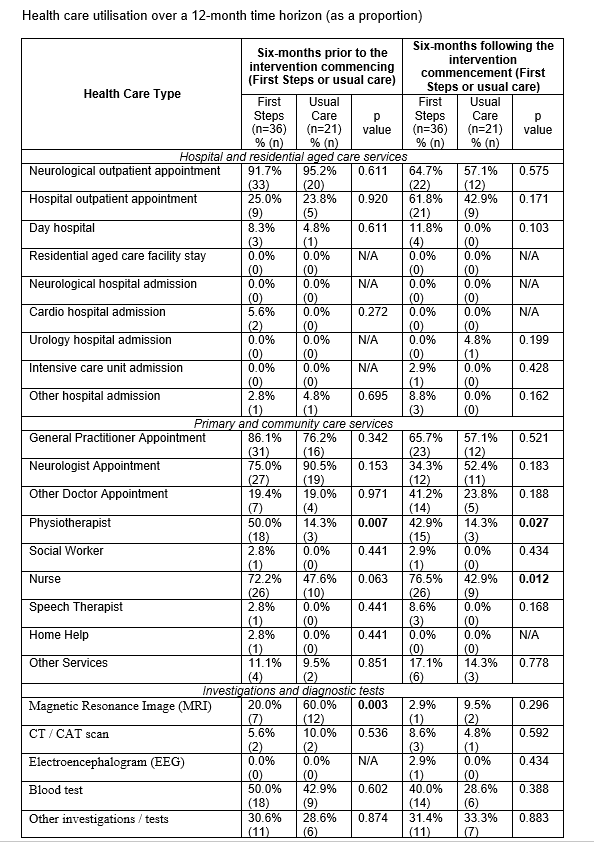

Supplement: sj-docx-1-cre-10.1177_02692155231210969 - Supplemental material for A programme evaluation of ‘First Steps’: A peer-conceived, developed and led self-management intervention for people after a Parkinson's diagnosis [file sj-docx-1-cre-10.1177_02692155231210969.docx]
